# Dynamics of Hydration Water Plays a Key Role in Determining the Binding Thermodynamics of Protein Complexes

**DOI:** 10.1038/s41598-017-09466-w

**Published:** 2017-08-18

**Authors:** Song-Ho Chong, Sihyun Ham

**Affiliations:** Department of Chemistry, Sookmyung Women’s University, Cheongpa-ro 47-gil 100, Yongsan-Ku, Seoul, 04310 Korea

## Abstract

Interfacial waters are considered to play a crucial role in protein–protein interactions, but in what sense and why are they important? Here, using molecular dynamics simulations and statistical thermodynamic analyses, we demonstrate distinctive dynamic characteristics of the interfacial water and investigate their implications for the binding thermodynamics. We identify the presence of extraordinarily slow (~1,000 times slower than in bulk water) hydrogen-bond rearrangements in interfacial water. We rationalize the slow rearrangements by introducing the “trapping” free energies, characterizing how strongly individual hydration waters are captured by the biomolecular surface, whose magnitude is then traced back to the number of water–protein hydrogen bonds and the strong electrostatic field produced at the binding interface. We also discuss the impact of the slow interfacial waters on the binding thermodynamics. We find that, as expected from their slow dynamics, the conventional approach to the water-mediated interaction, which assumes rapid equilibration of the waters’ degrees of freedom, is inadequate. We show instead that an explicit treatment of the extremely slow interfacial waters is critical. Our results shed new light on the role of water in protein–protein interactions, highlighting the need to consider its dynamics to improve our understanding of biomolecular bindings.

## Introduction

Water is an active and indispensable component of cells. Understanding its versatile roles in determining the structure and dynamics of biomolecules and mediating their interactions is of fundamental importance^1–3^. The versatility of water in biological contexts arises from its ability to alter its characteristics depending on its interaction with biomolecules. For example, the DNA sequence-dependent behavior of hydration water serves as a sequence-specific “hydration fingerprint”^4^; changes in water dynamics during binding of a substrate to an enzyme play a vital role in protein–ligand recognition^5^; and the non-bulk behavior of water inside the translocon strongly affects the partitioning of hydrophobic segments from the translocon to the membrane^6^. However, although our understanding of the behavior of hydration water around biomolecules has advanced significantly in recent years^7–14^, it remains a challenge to elucidate the extent to which water molecules located between two biomolecules are modified through concurrent interactions with the two binding surfaces and how such altered water molecules in turn affect the binding affinity.

In this connection, it has been suggested that water-mediated contacts substantially complement direct protein–protein contacts, providing an additional layer of biomolecular recognition^15, 16^. The necessity of an explicit treatment of interfacial water molecules to properly describe such water-mediated interactions has also been noted^17^. Indeed, recent computational studies have reported on the relevance of explicitly handling “key” interfacial waters in protein–protein interaction^18^ and protein–ligand binding^19^: for example, including two, rather than all, interfacial water molecules was crucial to correctly obtaining the trends observed in mutation effects on protein–protein binding affinity^18^; in another study, explicitly taking into account interfacial water molecules ranging in number (*N*_wat_) from 30 to 70 significantly improved the correlation with the experimental binding affinities for four different systems, where the optimum value of *N*_wat_ depended on the specific system^19^. What, however, distinguishes those key interfacial water molecules from others? Do any distinctive characteristics of the interfacial water emerge upon protein–protein complex formation?

In this paper, we investigate the dynamic and thermodynamic features of interfacial water in the barnase–barstar complex^15^. This is a well-studied paradigm for protein–protein interactions and is also an ideal system for analyzing the interfacial water because X-ray measurements indicate the presence of waters filling the gap between the binding surfaces^15, 20^. We perform molecular dynamics simulations to explore dynamic characteristics of the interfacial water. We focus on the rearrangements of hydrogen bonds, which are the most important protein–water interaction because the protein–protein binding surfaces comprise mainly polar and charged residues^21^. We then conduct statistical thermodynamic analyses to rationalize the dynamic characteristics of the interfacial water. Finally, we discuss the impact of the interfacial water dynamics on the protein–protein binding affinity. We find that the conventional approach to the water-mediated interaction, which assumes the time-scale separation between the protein and hydration water dynamics, fails owing to the extremely slow dynamics exhibited by the interfacial waters. We show instead that an explicit treatment of those slow waters as an integral part of biomolecules is critical. Thereby, we would like to shed new light on the role of water in protein–protein interactions based on a dynamic view point.

## Methods

### Molecular dynamics simulations

**Figure 1:**
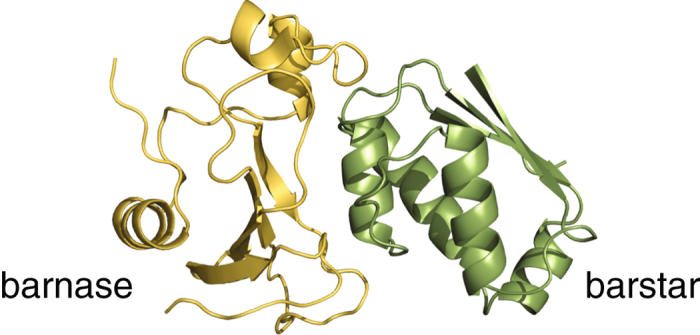
Structure of the barnase–barstar complex.

We conducted explicit-water molecular dynamics simulations for the barnase–barstar complex (Fig. 1) and for the free barnase and barstar proteins. The initial complex structure was modeled based on the X-ray structure (PDB ID: 1 BRS^15^) as detailed in ref. 22. The starting structures of the free barnase and barstar simulations were taken from their NMR structures (1 BNR^23^ and 1 BTA^24^, respectively). The complex structure was solvated by 23,477 waters and neutralized by 4 counter Na^+^ ions in a cubic box of the initial side length 95.4 Å; the free barnase (barstar) was solvated by 14,346 (8,397) waters and neutralized by 2 Cl^−^ (6 Na^+^) ions in a cubic box of the initial size 81.7 Å (69.5 Å). All the simulations were performed using the AMBER14 suite^25^ with the FF99SB force field^26^ for proteins and the TIP3P model^27^ for water. The temperature and pressure were maintained at *T* = 300 K and *P* = 1 bar using the Berendsen’s method^28^. We adopted the same simulation procedures as described in ref. 22, and three independent 1 *μ*s production runs were performed for each system starting from different random initial velocities.

We also conducted pure-water simulations to obtain dynamical quantities for bulk water. Three independent 100 ns simulations were performed at *T* = 300 K and *P* = 1 bar with 2,539 waters.

### Hydrogen-bond rearrangement dynamics

**Figure 2:**
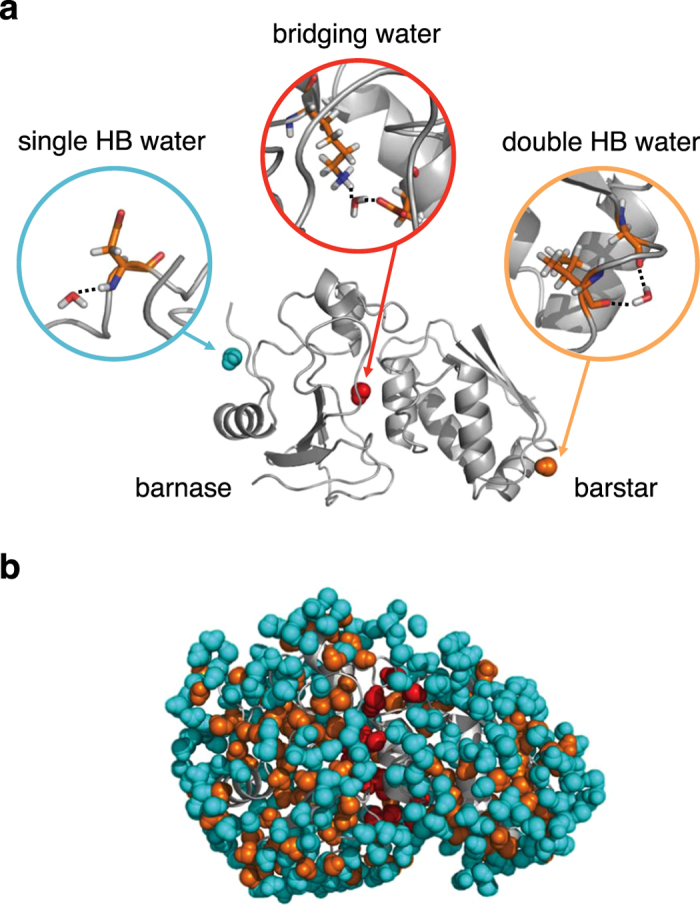
(**a**) Illustration of single HB, double HB, and bridging waters; dotted lines denote hydrogen bonds. (**b**) Snapshot of the barnase–barstar complex structure with hydration water, showing the distribution of single HB water (cyan), double HB water (orange), and bridging water (red).

We analyzed the hydrogen-bond time-correlation function, which quantifies the extent to which hydrogen bonds found at time *t* = 0 survive to subsequent times *t* ^29^, to investigate hydrogen-bond rearrangements between protein and hydration water. A hydrogen bond is considered formed when the water oxygen is located within 3.5 Å from heavy atoms in a protein. The hydration water is classified as follows (see Fig. 2a for an illustration). A water molecule forming a single hydrogen bond to a protein is referred to as single HB water. The locations of single HB waters in a simulation snapshot are indicated by cyan spheres in Fig. 2b, and their average number (±standard deviation) computed from the whole simulation trajectories for the complex is 325.7 ± 13.7 (Table 1 summarizes the number of water molecules and the number of water–protein hydrogen bonds). A water molecule making two or more hydrogen bonds to a protein is termed double HB water: the positions of double HB waters in a snapshot are shown by orange spheres in Fig. 2b. There are 133.0 ± 8.0 double HB waters in the system, and the average number of hydrogen bonds to a protein is 2.4 ± 0.1. Finally, a water molecule forming concurrent hydrogen bonds with two proteins is called bridging water. By definition, bridging waters are present only at the interface between two proteins (red spheres in Fig. 2b). We find 19.6 ± 3.0 bridging waters located at the interface, with the average number of water–protein hydrogen bonds being 2.9 ± 0.2.

**Table 1:** Average number of waters and water–protein hydrogen bonds^*a*^.

	Single HB water	Double HB water	Bridging water
# of waters	325.7 ± 13.7	133.0 ± 8.0	19.6 ± 3.0
# of H-bonds	1	2.4 ± 0.1	2.9 ± 0.2

^*a*^Average ± standard deviation.

We investigate the hydrogen-bond time-correlation function defined by

1 $${C}_{{\rm{HB}}}^{\alpha }(t)=\frac{\langle {C}_{{\rm{HB}},{\rm{indiv}}}^{\alpha }(t)\rangle }{\langle {C}_{{\rm{HB}},{\rm{indiv}}}^{\alpha }\mathrm{(0)}\rangle }$$

with the individual contributions ${C}_{\mathrm{HB},\mathrm{indiv}}^{\alpha }(t)$. Here, *α* refers to the type of water, and the brackets denote the average over water molecules of this type. For bulk and single HB water, ${C}_{{\rm{HB}},{\rm{indiv}}}^{\alpha }(t)$ is 1 if a hydrogen bond found at time 0 survives at time *t*. For double and bridging water, ${C}_{{\rm{HB}},{\rm{indiv}}}^{\alpha }(t)$ is 1 if two or more hydrogen bonds found at time *t* = 0 survive at time *t*.

### Trapping free energy

**Figure 3:**
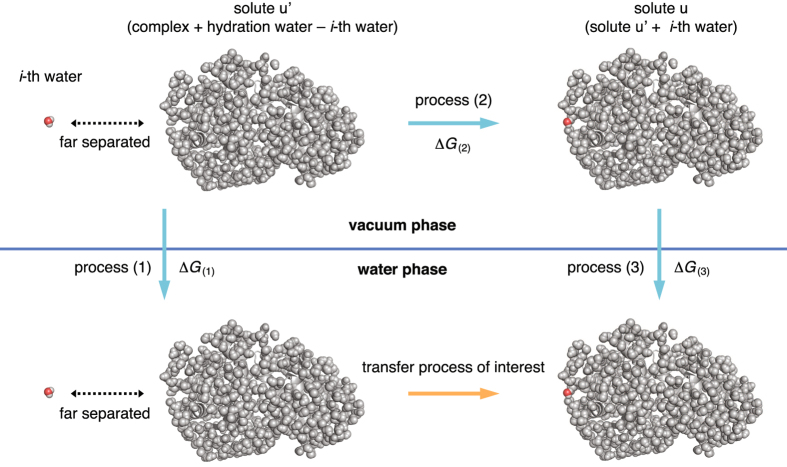
Thermodynamic cycle for obtaining the trapping free energy. Process (1) is the separate solvation processes of the *i*-th water molecule and the solute *u*′ (barnase–barstar complex + hydration water − *i*-th water molecule). Process (2) is the transfer process of the *i*-th water molecule to a specific position and orientation around the solute *u*′ in vacuum. Process (3) is the solvation process of the solute *u* (=solute *u*′ + *i*-th water molecule). From the Gibbs free energy changes associated with these processes, the transfer free energy of interest can obtained as −Δ*G*_(1)_ + Δ*G*_(2)_ + Δ*G*_(3)_.

We introduce the trapping free energies of individual hydration waters to quantify how strongly they are bound to the protein surface. The trapping free energy refers to the reversible work (i.e., the potential of mean force) for transferring a water molecule from an infinite separation to a specific position and orientation relative to the protein–protein complex. We consider here the transfer process to a fixed position and orientation relative to the solute for two reasons. First, this allows us to compute the trapping free energies of individual hydration waters solely based on the simulation snapshots such as the one presented in Fig. 2b. Second, we are interested in whether the trapping free energies so computed at time *t* = 0 serve as a descriptor of the degree of retardation of the subsequent (*t* > 0) dynamics of individual water molecules. The thermodynamic cycle shown in Fig. 3 is used to obtain this quantity; in this cycle, we consider the transfer of the *i*-th water molecule to a specific position and orientation around the solute *u*, which includes the hydration water molecules of interest (e.g., all the waters displayed in Fig. 2b). The solute from which the *i*-th water molecule is excluded is denoted as *u*′.

Process (1) in Fig. 3 is the independent solvation processes of the *i*-th water molecule and the solute *u*′; hence, the associated Gibbs free energy change is given by ${\rm{\Delta }}{G}_{\mathrm{(1)}}={G}_{{\rm{water}}}^{{\rm{solv}}}+{G}_{u^{\prime} }^{{\rm{solv}}}$, which consists of the solvation free energies of a single water molecule (${{G}}_{{\rm{water}}}^{{\rm{solv}}}$) and of the solute *u*′ (${{G}}_{u^{\prime} }^{{\rm{solv}}}$). In process (2), the *i*-th water molecule is transferred to a specific position and orientation around the solute *u*′ from an infinite separation in vacuum. The reversible work required for this process is given by the interaction energy ${E}_{u^{\prime} -i}$ between the solute *u*′ and the *i*-th water molecule, ${\rm{\Delta }}{G}_{\mathrm{(2)}}={E}_{u^{\prime} -i}$. Process (3) is the solvation of the solute *u*(=*u*′ + *i*), and we obtain ${\rm{\Delta }}{G}_{\mathrm{(3)}}={G}_{u(=u^{\prime} +i)}^{{\rm{solv}}}$. From the thermodynamic cycle, one obtains the trapping free energy from –Δ*G*_(1)_ + Δ*G*_(2)_ + Δ*G*_(3)_; that is,

2 $${G}_{i}^{{\rm{trap}}}={E}_{u^{\prime} -i}+{G}_{u(=u^{\prime} +i)}^{{\rm{solv}}}-{G}_{u^{\prime} }^{{\rm{solv}}}-{G}_{{\rm{water}}}^{{\rm{solv}}}$$

We computed the interaction energy ${E}_{u^{\prime} -i}$ from the force field, whereas the solvation free energy ${{G}}_{u}^{{\rm{solv}}}$ is obtained using the 3D-RISM theory^30^, whose details are presented in Supplementary Methods. An efficient method for computing the contribution ${{G}}_{u(=u^{\prime} +i)}^{{\rm{solv}}}-{G}_{u^{\prime} }^{{\rm{solv}}}$ which is based on the atomic decomposition of the solvation free energy^31, 32^ is also provided there. The trapping free energies for the hydration waters surrounding free barnase and barstar proteins can be obtained in a similar manner.

Recently, several computational methods have been developed for evaluating thermodynamic functions of individual hydration waters^33–41^. However, these methods typically demand performing additional distinct simulations. For example, the application of the inhomogeneous solvation theory^33–35^ requires conducting simulations in which restrains are added on protein atoms to sample waters’ positions and orientations for a given protein conformation. Further complications in analysis will arise when hydration waters exchange with bulk waters during those additional simulations. On the other hand, our computational method for the trapping free energy that employs the integral-equation theory (3D-RISM) is applicable solely based on snapshots taken from unrestrained equilibrium simulations, and it is in this sense more computationally efficient.

### Standard binding free energy

#### Conventional approach

The statistical thermodynamic expression for the standard binding free energy is given by refs 22 and 42

3 $${\rm{\Delta }}{G}_{{\rm{bind}}}^{0}=\overline{{\rm{\Delta }}{f}_{u}}-T({\rm{\Delta }}{S}_{{\rm{config}}}+{\rm{\Delta }}{S}_{{\rm{ext}}})$$

Here, Δ*X* denotes the change in *X* upon complex formation from two free proteins (labeled 1 and 2), Δ*X* = *X*_complex_ − (*X*_1_ + *X*_2_); ${f}_{u}={E}_{u}+{G}_{u}^{{\rm{solv}}}$ comprises the gas-phase energy (*E*_*u*_) and the solvation free energy (${{G}}_{u}^{{\rm{solv}}}$) of the solute *u* (here, *u* refers to the complex or one of the two free proteins and excludes hydration waters); the bar denotes the average over the simulated configurations; *S*_config_ is the configurational entropy associated with the solute’s internal degrees of freedom; and Δ*S*_ext_ is the entropy change originating from the reduction in the external (positional and orientational) degrees of freedom upon complex formation. Δ*S*_ext_ carries the standard-state dependence, which is chosen here to be the one of the standard concentration (1 M).

We computed the gas-phase energy *E*_*u*_ from the force field adopted in the simulations. (*E*_*u*_ for free proteins represents only the intra-protein energy, but *E*_*u*_ for the complex includes the inter-protein interaction energy as well). For the solvation free energy ${{G}}_{u}^{{\rm{solv}}}$, we employed the 3D-RISM theory^30^ (see Supplementary Methods). For the configurational entropy *S*_config_, we used an energetic approach^43, 44^ that expresses *S*_config_ in terms of the statistical properties of *f*_*u*_. In particular, when the probability distribution *W*(*f*_*u*_) of *f*_*u*_ is Gaussian, the following holds:

4 $$T\,{S}_{{\rm{config}}}=\frac{1}{2{k}_{{\rm{B}}}T}\,\overline{\delta \,{f}_{u}^{2}}$$

where *k*_B_ is Boltzmann’s constant, and $\delta \,{f}_{u}={f}_{u}-\overline{{f}_{u}}$. For the external entropy Δ*S*_ext_, we use the estimate *T*Δ*S*_ext_ = −6.8 ± 0.1 kcal/mol for the barnase–barstar complex, which was computed in ref. 22. by extending the energetic approach to the binding process and is close to the value reported in ref. 45.

#### Explicit inclusion of the water molecules of interest

A statistical thermodynamic formulation of the binding free energy which allows one to explicitly include certain solvent molecules was also derived in ref. 42. In essence and using our notation, what is required is to replace ${f}_{u}={E}_{u}+{G}_{u}^{{\rm{solv}}}$, which appears in equation (3) for ${\rm{\Delta }}{G}_{{\rm{bind}}}^{0}$, with

5 $${f}_{u}={E}_{u}+{G}_{u}^{{\rm{solv}}}-n{G}_{{\rm{water}}}^{{\rm{solv}}}$$

In this expression, the solute *u* now explicitly includes the water molecules of interest (e.g., *E*_*u*_ now contains interactions with and among those water molecules), *n* is the number of water molecules included, and ${{G}}_{{\rm{water}}}^{{\rm{solv}}}$ is the single water molecule’s solvation free energy. *S*_config_ then needs to be evaluated by combining equations (4) and (5).

## Results and Discussion

### Hydrogen-bond rearrangement dynamics

We study the dynamic and thermodynamic features of the hydration water surrounding the barnase–barstar complex by conducting molecular dynamics simulations and statistical thermodynamic analyses. In particular, we aim to uncover the distinctive characteristics of the interfacial water between two proteins that emerge upon complex formation. This is done by contrasting the dynamics of the interfacial water with that of the hydration water surrounding free proteins; for this purpose, we also perform simulations and analyses for the free barnase and barstar proteins. We focus on the rearrangements of hydrogen bonds, which are the most important protein–water interactions because of the largely hydrophilic nature of the protein–protein binding surfaces^21^.

**Figure 4:**
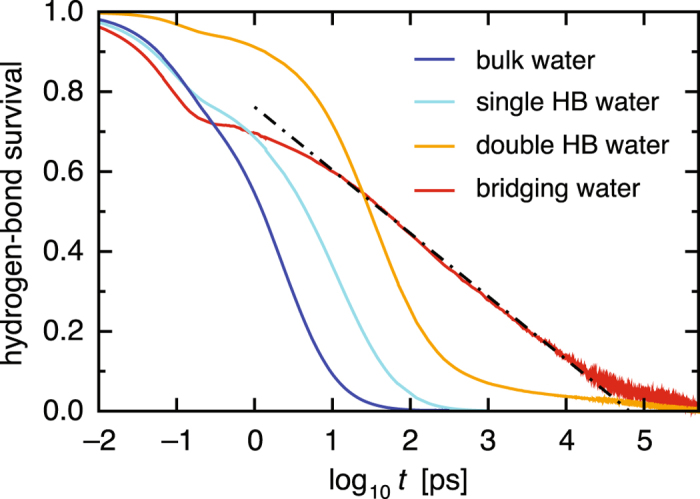
Hydrogen-bond time-correlation functions for bulk water (blue), single HB water (cyan), double HB water (orange), and bridging water (red) versus the logarithmic time axis. Dashed-dotted line denotes the fit by a logarithmic function.

Figure 4 shows the hydrogen-bond time-correlation functions, which quantify the extent to which hydrogen bonds found at time *t* = 0 remain at subsequent times *t*. For water molecules making a single hydrogen bond to a protein (referred to as single HB water; see Fig. 2 and Table 1), we observe profound slowing down of the relaxation dynamics compared to those of bulk water (see Table 2 for a comparison of the average relaxation times). For water molecules making two or more hydrogen bonds to a protein (double HB water), the hydrogen-bond rearrangement is even slower. For bridging water molecules, that is, interfacial water molecules making concurrent hydrogen bonds with two proteins, the relaxation is extraordinarily slow (~1,000 times slower than the relaxation in bulk water). Furthermore, the relaxation curve is anomalous, exhibiting a logarithmic decay over three orders of magnitude in time.

**Table 2:** Average relaxation time and trapping free energy^*a*^.

	Bulk water	Single HB water	Double HB water	Bridging water
Relaxation time 〈*τ*_*i*_〉^*b*^	5.1 ± 1.6	28.1 ± 10.1	163.3 ± 83.7	4954 ± 4418
(〈log_10_ *τ*_*i*_〉)^*c*^	(0.69 ± 0.13)	(1.42 ± 0.16)	(2.17 ± 0.20)	(3.52 ± 0.39)
Trapping free energy^*d*^	0	−4.5 ± 3.4	−8.0 ± 4.1	−12.5 ± 4.7

^*a*^Average ± standard deviation; ^*b*^Relaxation time in ps computed with individual relaxation times *τ*_*i*_; ^*c*^Statistics computed with log_10_ *τ*_*i*_; ^*d*^Trapping free energy in kcal/mol.

### Thermodynamic–dynamic relationship diagram

**Figure 5:**
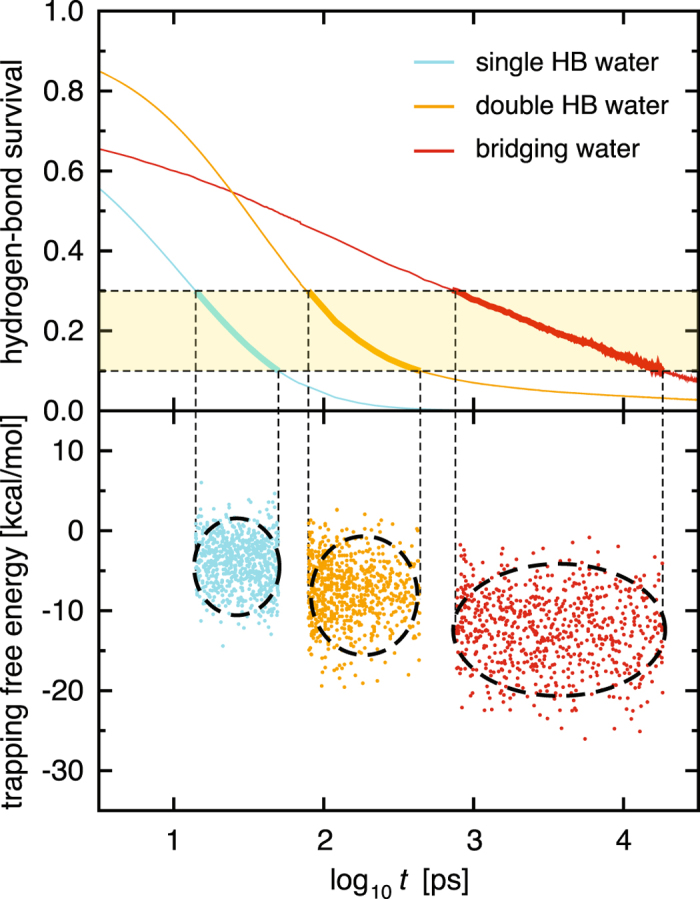
Construction of the thermodynamic–dynamic relationship diagram. Upper panel: Hydrogen-bond time-correlation functions for single HB water (cyan), double HB water (orange), and bridging water (red) taken from Fig. 4, focusing on the time regime where the functions decay from 0.3 to 0.1 (light yellow). Lower panel: Scatter plots of the hydrogen-bond survival times (*τ*_*i*_) and trapping free energies (${{G}}_{i}^{{\rm{trap}}}$) of individual water molecules. Centers of ellipsoids are determined by the averages, and the width and hight are determined by 3.6 *σ* (where *σ* is the standard deviation) along each axis.

To rationalize the slow relaxations of hydration waters, we conducted statistical thermodynamic analyses. We focused on the long-time region where the time-correlation functions decay from 0.3 to 0.1 (light yellow region in the upper panel of Fig. 5) and extracted the water molecules contributing to the relaxation there by examining the hydrogen-bond survival times (*τ*_*i*_) of individual molecules. For each of those water molecules, we computed the trapping free energy (${{G}}_{i}^{{\rm{trap}}}$) using the simulation snapshot at *t* = 0. The trapping free energy can be considered as the effective potential characterizing how strongly each water molecule is captured by the biomolecular surface: a more negative trapping free energy means that a water molecule is more stable near the protein complex than in the bulk, and hence, is more favorably “trapped” by the protein complex. The lower panel of Fig. 5 shows scatter plots of the relaxation times and trapping free energies of individual water molecules. (The average relaxation times and trapping free energies listed in Table 2 are obtained from these plots. Since the distribution of the individual waters’ relaxation times *τ*_*i*_ is well represented on the logarithmic axis as shown in the lower panel of Fig. 5, Table 2 also provides the statistics computed with log_10_ *τ*_*i*_). The resulting “thermodynamic–dynamic relationship diagram” clearly illustrates that the trapping free energy (${{G}}_{i}^{{\rm{trap}}}$) at *t* = 0 serves as a good descriptor of the degree of retardation (*τ*_*i*_) of the subsequent dynamics of hydration water.

**Figure 6:**
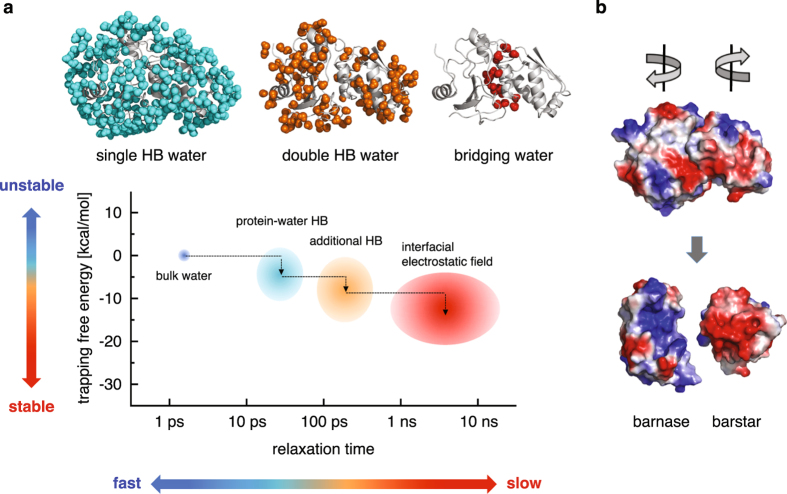
(**a**) Schematic representation of the thermodynamic–dynamic relationship diagram of hydration water. Here, the lower panel from Fig. 5 is schematically redrawn for single HB water (cyan), double HB water (orange), and bridging water (red), along with the position for the bulk water (blue). (**b**) Protein surfaces color-coded with the charge distribution (blue and red for positively and negatively charged regions, respectively).

Thermodynamic–dynamic relationship diagram is presented schematically in Fig. 6a. Single HB water exhibits slower dynamics than bulk water because it is more stable near the protein surface, which in turn reflects the fact that the hydrogen bond between water and protein is stronger than the one between waters. The even slower dynamics of double HB water can be understood similarly; further stabilization originates from an additional water–protein hydrogen bond. Why, then, is bridging water, which has the comparable number of hydrogen bonds with proteins as double HB water (Table 1), more strongly trapped than double HB water? We also notice here that the dynamic, as well as thermodynamic, characteristics of single and double HB water molecules are nearly the same, irrespective of whether they are placed near the isolated monomer or protein–protein complex (Supplementary Figs S1 and S2). The emergence of the “red region” for bridging water in the diagram (Fig. 6a) thus arises solely from the formation of the protein–protein interface. Is there any special factor that is effective only at the interface?

We notice in this regard the electrostatic complementarity of the barnase–barstar binding surfaces (Fig. 6b), which creates a strong electrostatic field that is exerted on the interfacial water. Indeed, we find that the magnitude of the electrostatic field is stronger and the water’s dipole vector is more oriented along the electrostatic field for bridging water than for single and double HB water (Supplementary Fig. S3). Thus, whereas essentially no change in the hydration water dynamics is observed in the non-interfacial region before and after the binding (Supplementary Figs S1 and S2), the strong electrostatic field created at the binding interface produces an extra stabilizing factor for bridging water, causing it to exhibit extremely slow (nanosecond timescale) hydrogen-bond relaxations (Table 2). It would be interesting to investigate how the transition in the hydration water dynamics occurs during the binding process, but for this purpose, one needs to perform spontaneous binding simulations. The trapping free energy introduced in the present work serves as a valuable quantity not only to characterize the formation of the binding interface from the water’s perspective, but also to discuss how and whether the hydration water is rearranging to go from the unbound protein to bound complex. While the barnase–barstar complex studied here is known to be a system in which the interfacial waters are particularly immobile^46^, we anticipate the emergence of the extremely slow water relaxations to be a generic feature of hydrophilic protein–protein interfaces because electrostatic complementarity of the binding surfaces has been observed in numerous protein complexes^47, 48^.

### “Conventional” binding thermodynamics

Among the terms that contribute to ${\rm{\Delta }}{G}_{{\rm{bind}}}^{0}$ (see equation (3)), the quantity ${\rm{\Delta }}{f}_{u}={\rm{\Delta }}{E}_{u}+{\rm{\Delta }}{G}_{u}^{{\rm{solv}}}$ plays a special role for two reasons. First, it is generally difficult to compute the configurational (Δ*S*_config_) and external (Δ*S*_ext_) entropies for complex macromolecules such as proteins. Second, Δ*S*_config_ and Δ*S*_ext_ are usually negative upon complex formation and thus make unfavorable positive contributions to ${\rm{\Delta }}{G}_{{\rm{bind}}}^{0}$; hence, the driving force for binding must originate from Δ*f*_*u*_. Indeed, Δ*f*_*u*_ is the central quantity, termed the effective binding free energy^49^, in computational approaches to biomolecular bindings such as the molecular-mechanics Poisson-Boltzmann surface area (MM-PBSA) method^50–52^.

We computed $\overline{{\rm{\Delta }}{f}_{u}}$ by averaging Δ*f*_*u*_ over the simulated protein conformations. (Our approach is referred to as the three-trajectory approach because we conducted separate computations for the complex and for two free proteins. Numerical values for the binding thermodynamics shall be reported with standard errors computed based on the respective independent trajectories of the complex and free proteins and on the rule of error propagation). The energetic contributions (Δ*E*_*u*_) were calculated directly from the force field, and the solvation contributions (${\rm{\Delta }}{G}_{u}^{{\rm{solv}}}$) were computed using the 3D-RISM theory (see Supplementary Methods). We obtained $\overline{{\rm{\Delta }}{f}_{u}}=+25.7\pm 2.6$ kcal/mol; this result leads to an unphysical positive value of ${\rm{\Delta }}{G}_{{\rm{bind}}}^{0}$, which is in obvious disagreement with the experimental observation (${\rm{\Delta }}{G}_{{\rm{bind}}}^{0}=-18.9$ kcal/mol)^53^. Interestingly, positive effective binding free energy has also been reported based on the MM-PBSA calculations for the barnase–barstar complex: $\overline{{\rm{\Delta }}{f}_{u}}=+14$ kcal/mol in ref. 54 and $\overline{{\rm{\Delta }}{f}_{u}}=+3.6$ kcal/mol in ref. 55. (The difference in these values may originate from the use of the one-trajectory approach in the MM-PBSA calculations, in which both the complex and monomer configurations were taken from simulations of the complex; the use of different force fields; and the use of different approximations for the solvation free energy).

### Basic assumption behind the conventional approach

At this point, we critically examine the basic assumption behind the expression (3) for the standard binding free energy. To simplify the discussion, we work in the canonical ensemble and ignore the external entropy, which would not alter the essential point here. We start from the configuration integral, the potential part of the partition function, for a solute-solvent system:

6 $${Z}_{{\rm{tot}}}=\int \,d{{\bf{r}}}_{u}\,\int \,d{{\bf{r}}}_{v}\,{e}^{-\beta [{E}_{u}({{\bf{r}}}_{u})+{E}_{uv}({{\bf{r}}}_{u},{{\bf{r}}}_{v})+{E}_{v}({{\bf{r}}}_{v})]}$$

Here, **r**_*u*_ and **r**_*v*_ collectively denote the solute and solvent degrees of freedom, respectively; *β* = 1/(*k*_B_ *T*) is the inverse temperature; and *E*_*u*_, *E*_*uv*_, and *E*_*v*_ are the solute energy, solute-solvent interaction energy, and solvent-solvent interaction energy, respectively. *Z*_tot_ is the principal object in free energy simulations: the change in the free energy *F*_tot_ = −*k*_B_ *T* log *Z*_tot_, e.g., upon mutation, is computed from simulations in which both the solute and solvent degrees of freedom are explicitly handled. However, equation (6) does not serve as a basis of equation (3): for example, by introducing the probability distribution ${p}_{{\rm{tot}}}({{\bf{r}}}_{u},{{\bf{r}}}_{v})={e}^{-\beta {E}_{{\rm{tot}}}({{\bf{r}}}_{u},{{\bf{r}}}_{v})}/{Z}_{{\rm{tot}}}$ with *E*_tot_ = *E*_*u*_ + *E*_*uv*_ + *E*_*v*_, the entropy that naturally arises from equation (6) is ${S}_{{\rm{tot}}}=-{k}_{{\rm{B}}}\,\int \,d{{\bf{r}}}_{u}\,\int \,d{{\bf{r}}}_{v}\,{p}_{{\rm{tot}}}({{\bf{r}}}_{u},{{\bf{r}}}_{v})\,\mathrm{log}\,{p}_{{\rm{tot}}}({{\bf{r}}}_{u},{{\bf{r}}}_{v})$, and it is non-trivial to partition the solute and solvent terms from this total entropy. This is in contrast to equation (3) where the solute (*S*_config_) and solvent (contained in ${G}_{u}^{{\rm{solv}}}$) entropies are separated.

To arrive at equation (3) from equation (6), one has to resort to a pre-averaging of solvent degrees of freedom. For a given solute configuration **r**_*u*_, this pre-averaging can be performed as

7 $${e}^{-\beta {G}_{u}^{{\rm{solv}}}({{\bf{r}}}_{u})}=\frac{1}{{Z}_{v}}\,\int \,d{{\bf{r}}}_{v}\,{e}^{-\beta [{E}_{uv}({{\bf{r}}}_{u},{{\bf{r}}}_{v})+{E}_{v}({{\bf{r}}}_{v})]}$$

in terms of the solute-configuration dependent solvation free energy ${{G}}_{u}^{{\rm{solv}}}({{\bf{r}}}_{u})$. Here, ${Z}_{v}=\int \,d{{\bf{r}}}_{v}\,{e}^{-\beta {E}_{v}({{\bf{r}}}_{v})}$ is the configuration integral for the pure solvent. Now, the configuration integral after the pre-averaging of the solvent degrees of freedom is given by

8 $$Z\equiv {Z}_{{\rm{tot}}}/{Z}_{v}=\int \,d{{\bf{r}}}_{u}\,{e}^{-\beta {f}_{u}({{\bf{r}}}_{u})}$$

with ${f}_{u}={E}_{u}+{G}_{u}^{{\rm{solv}}}$. By introducing $p({{\bf{r}}}_{u})={e}^{-\beta {f}_{u}({{\bf{r}}}_{u})}/Z$, the associated entropy is given by $-{k}_{{\rm{B}}}\,\int \,d{{\bf{r}}}_{u}\,p({{\bf{r}}}_{u})\,\mathrm{log}\,p({{\bf{r}}}_{u})$, which is the defining equation for the solute configurational entropy *S*_config_. It is therefore clear that equation (3) is based on the pre-averaging of solvent degrees of freedom (see refs 22 and 42 for a complete derivation of equation (3) from equation (8)). By the “conventional” approach, we mean the one that is based on this pre-averaging, and do not refer to specific methods such as PBSA and 3D-RISM.

In practical applications of the conventional approach, one takes only the protein conformations from simulation trajectories, replacing all the explicit water molecules by the equilibrium continuum model (PBSA) or molecular distribution function (3D-RISM). Such a treatment is usually justified because of the timescale separation between the typical water dynamics (picoseconds) and the protein conformational motions (nanoseconds)^16^, i.e., because of the rapid equilibration of surrounding waters. However, the extreme slowness of the bridging-water relaxation may invalidate such a naive treatment of the water at biomolecular interfaces, and we conjectured that this might be the origin of the unphysical positive value of $\overline{{\rm{\Delta }}{f}_{u}}$.

### Explicit inclusion of bridging water

We therefore investigated the impact of explicit inclusion of the slow bridging waters. For this purpose, we not only take the protein configurations from the simulation trajectories for the complex, but also bridging waters located at the interface: the number (*n*) of bridging waters depends on the simulation snapshot, and its average value is 19.6 ± 3.0 (Table 1). Now, those bridging waters are considered as a structural part of the complex, and we apply equation (5) to compute *f*_*u*_ for the complex. We obtain a negative value $\overline{{\rm{\Delta }}{f}_{u}}=-34.2\pm 2.1$ kcal/mol, which now serves as the driving force for binding. This result indicates the necessity of considering the dynamics of the interfacial water in the binding thermodynamics.

To support our explicit inclusion of bridging waters through a comparison with experiment, we computed the binding free energy ${\rm{\Delta }}{G}_{{\rm{bind}}}^{0}$. To this end, we need to estimate the configurational (Δ*S*_config_) and external (Δ*S*_ext_) entropy contributions. For the configurational entropy, we used an energetic approach^43, 44^ that expresses Δ*S*_config_ in terms of the fluctuations in *f*_*u*_. In particular, when the probability distribution *W*(*f*_*u*_) of *f*_*u*_ is Gaussian, *S*_config_ is simply given by the mean-squared fluctuations of *f*_*u*_ (see equation (4)). Indeed, *W*(*f*_*u*_) of the barnase–barstar complex with bridging water is well approximated by Gaussian, as well as that of the free barnase and barstar proteins (Supplementary Fig. S4), from which *T*Δ*S*_config_ = −4.5 ± 18.5 kcal/mol is obtained. For the external entropy Δ*S*_ext_, we use the estimate *T*Δ*S*_ext_ = −6.8 ± 0.1 kcal/mol, which was obtained using the method developed in ref. 22 and is close to the value reported in ref. 45. Combining all these results, we obtain ${\rm{\Delta }}{G}_{{\rm{bind}}}^{0}=-22.9\pm 18.6$ kcal/mol, which is in reasonable accord with experiment (−18.9 kcal/mol)^53^. (As can be inferred from the numerical values presented above, the large standard error for ${\rm{\Delta }}{G}_{{\rm{bind}}}^{0}$ originates from that for *T*Δ*S*_config_; indeed, the protein configurational entropy is known as the most difficult thermodynamic parameter to estimate).

## Conclusions

Water molecules are ubiquitously found at the interfaces between biomolecules, and it is often stated that the interfacial water must be considered as an integral part of biomolecules. The work presented here sheds new light on this statement based on the dynamic viewpoint. We demonstrate the emergence of “special” waters in the interfacial region that bridge two biomolecules through concurrent hydrogen bonds and exhibit extremely slow hydrogen-bond rearrangements. By analyzing the thermodynamic–dynamic relationship diagram, we find that the extremely slow nature of bridging water is due to not only the number of hydrogen bonds involved, but also the additional stabilization resulting from the strong electrostatic field between the binding surfaces of electrostatic complementarity. The role of such slow interfacial waters in determining the binding affinity cannot be described using the conventional approach to the water-mediated interaction, which assumes rapid equilibration of the waters’ degrees of freedom. Indeed, we observe that a meaningful estimate of the binding affinity is achieved only with a unified treatment of both the biomolecules and the interfacial bridging water. Our work thus demonstrates the impact of the hydration dynamics on the protein–protein binding thermodynamics.

## Electronic supplementary material

Supplementary Information
