# Supplementary material for: Dynamics of Hydration Water Plays a Key Role in Determining the Binding Thermodynamics of Protein Complexes
Source: Sci Rep. 2017 Aug 18;7:8744. doi: 10.1038/s41598-017-09466-w (PMC5562873; doi:10.1038/s41598-017-09466-w)
Supplement: Supplementary file 1 — Supplementary Information [file 41598_2017_9466_MOESM1_ESM.pdf]

Supplementary Information for

Dynamics of Hydration Water Plays a Key Role in Determining the Binding Thermodynamics of Protein Complexes

Song-Ho Chong and Sihyun Ham*

Department of Chemistry, Sookmyung Women's University,

Cheongpa-ro 47-gil 100, Yongsan-Ku, Seoul 04310, Korea

Corresponding author:

*Sihyun Ham

**Address**	Department of Chemistry, Sookmyung Women's University
	Cheongpa-ro 47-gil 100, Yongsan-Ku, Seoul 04310, Korea
**Email**	sihyun@sookmyung.ac.kr
**Phone**	+82-2-710-9410
**Fax**	+82-2-2077-7321

Calculation method of solvation free energy

Integral-equation theory. To each of the simulated solute configurations, we applied the three-dimensional reference interaction site model (3D-RISM) theory[S1,S2] to compute the solvation free energy $G_u^{\rm solv}$. The 3D-RISM theory is an integral-equation theory based on statistical mechanics for obtaining the 3D distribution function $g_\gamma(\mathbf{r})$ of the water site γ at position $\mathbf{r}$ around the solute. (The solute here refers either to the barnase–barstar complex, barnase monomer, barstar monomer, or one of these protein systems plus explicitly-handled water molecules. In this case, there are two kinds of water: one that is explicitly considered as a part of the solute, and the other surrounding the solute implicitly handled through the distribution function.) In this theory, the distribution function is obtained by self-consistently solving the 3D-RISM equation

$$h_\gamma(\mathbf{r}) = \sum_{\gamma'} \int d\mathbf{r}'\, \chi_{\gamma\gamma'}(|\mathbf{r}-\mathbf{r}'|)\, c_{\gamma'}(\mathbf{r}') \tag{S1}$$

and the approximate closure relation

$$h_\gamma(\mathbf{r}) = \begin{cases} \exp[d_\gamma(\mathbf{r})] - 1 & \text{for} \quad d_\gamma(\mathbf{r}) \le 0 \\ d_\gamma(\mathbf{r}) & \text{for} \quad d_\gamma(\mathbf{r}) > 0 \end{cases} \tag{S2}$$

in which $d_\gamma(\mathbf{r}) = -u_\gamma(\mathbf{r})/(k_{\rm B}T) + h_\gamma(\mathbf{r}) - c_\gamma(\mathbf{r})$. Here $h_\gamma(\mathbf{r}) = g_\gamma(\mathbf{r}) - 1$ and $c_\gamma(\mathbf{r})$ are the total and direct correlation functions, respectively; $\chi_{\gamma\gamma'}(r)$ denotes the site-site water susceptibility function, treated as an input to the theory, which can be obtained either from simulations or integral-equation calculations for pure water; and $u_\gamma(\mathbf{r})$ is the solute-solvent interaction potential for a given solute configuration. We used the same numerical procedure as described in ref S2 to solve the above equations.

Solvation free energy can be computed from Kirkwood's charging formula,[S3]

$$G_u^{\rm solv} = \rho \sum_\gamma \int_0^1 d\lambda \int d\mathbf{r}\, g_\gamma(\mathbf{r};\lambda)\, \frac{\partial u_\gamma(\mathbf{r};\lambda)}{\partial \lambda} \tag{S3}$$

Here, λ is the charging parameter that gradually switches on the solute-solvent interaction from no interaction at $\lambda = 0$, $u_\gamma(\mathbf{r}; \lambda = 0)$, to the full interaction at $\lambda = 1$, $u_\gamma(\mathbf{r}; \lambda = 1) = u_\gamma(\mathbf{r})$. $g_\gamma(\mathbf{r}; \lambda)$ denotes the distribution function at the parameter λ, and can be calculated from the 3D-RISM theory using the interaction potential $u_\gamma(\mathbf{r}; \lambda)$.[S4]

Decomposition method. In computing the trapping free energy introduced in the main text (see eq 2), we need to evaluate the contribution $G^{\text{solv}}_{u(=u'+i)} - G^{\text{solv}}_{u'}$. Here, the difference between the solutes $u(= u'+i)$ and u' is in the presence/absence of the i-th water molecule of interest (see Figure 3 in the main text). To compute the trapping free energy for each of the hydration water molecules of a given solute configuration, we need to repeat the calculation of this term as many times as the number of those water molecules. This is computationally quite demanding since we find that there are, on average, 533 hydration water molecules surrounding the barnase–barstar complex.

To circumvent this computational problem, we introduce the following approximation:

$$G^{\text{solv}}_{u(=u'+i)} - G^{\text{solv}}_{u'} \approx G^{\text{solv}}_{i:\,u} \tag{S4}$$

Here, $G^{\text{solv}}_{i:\,u}$ is the contribution of the i-th water molecule to the solvation free energy G^{solv}_{u} of the solute that includes all the hydration water molecules of interest. As presented below, $G^{\text{solv}}_{i:\,u}$ for all the hydration water molecules of a given solute configuration can be computed from a single decomposition calculation of G^{solv}_{u}, which is computationally quite efficient.

The decomposition method of the solvation free energy[S4,S5] is based on the fact that the interaction potential $u_\gamma(\mathbf{r})$ between the water site γ and the solute atoms consists of the pairwise additive contributions, $u_\gamma(\mathbf{r}) = \sum_a u_{\gamma a}(|\mathbf{r} - \mathbf{r}_a|)$, centered at the solute atom a of position $\mathbf{r}_a$. Substituting this into the Kirkwood charging formula (eq S3) yields an exact atomic decomposition of the solvation free energy,[S4,S5]

$$G^{\text{solv}}_{u} = \sum_a G^{\text{solv}}_{a:\,u} \tag{S5}$$

Here, we used the notation $G^{\mathrm{solv}}_{a:u}$ to emphasize that it is the contribution from atom a in solute u. Once the atomic decomposition of G^{solv}_{u} is done, the contribution $G^{\mathrm{solv}}_{i:u}$ from the i-th water molecule can be obtained from $G^{\mathrm{solv}}_{i:u} = \sum_{a \in i} G^{\mathrm{solv}}_{a:u}$ in terms of the contributions from its constituent atoms, and this relation holds for all the hydration water molecules included in the solute u.

We examined the validity of the approximation given in eq S4 for the hydration water molecules of 30 barnase–barstar complex configurations, which were chosen with a 100 ns time interval from 3 independent 1 μs trajectories. We found that the Pearson correlation coefficient (R) between $G^{\mathrm{solv}}_{u(=u'+i)} - G^{\mathrm{solv}}_{u'}$ and $G^{\mathrm{solv}}_{i:u}$ is $R = 0.85$, indicating that $G^{\mathrm{solv}}_{i:u}$ serves as a good approximation of the term $G^{\mathrm{solv}}_{u(=u'+i)} - G^{\mathrm{solv}}_{u'}$.

References

(S1) Kovalenko, A. In *Molecular Theory of Solvation*; Hirata, F., Ed.; Kluwer Academic: Dordrecht, 2003; p 169.

(S2) Imai, T.; Harano, Y.; Kinoshita, M.; Kovalenko, A.; Hirata, F. A Theoretical Analysis on Hydration Thermodynamics of Proteins. *J. Chem. Phys.* **2006**, *125*, 024911.

(S3) Ben-Naim, A. *Molecular Theory of Solutions*; Oxford University Press: New York, 2006.

(S4) Chong, S.-H.; Ham, S. Atomic Decomposition of the Protein Solvation Free Energy and Its Application to Amyloid-Beta Protein in Water. *J. Chem. Phys.* **2011**, *135*, 034506.

(S5) Chong, S.-H.; Ham, S. Distinct Role of Hydration Water in Protein Misfolding and Aggregation Revealed by Fluctuating Thermodynamics Analysis. *Acc. Chem. Res.* **2015**, *48*, 956–965.

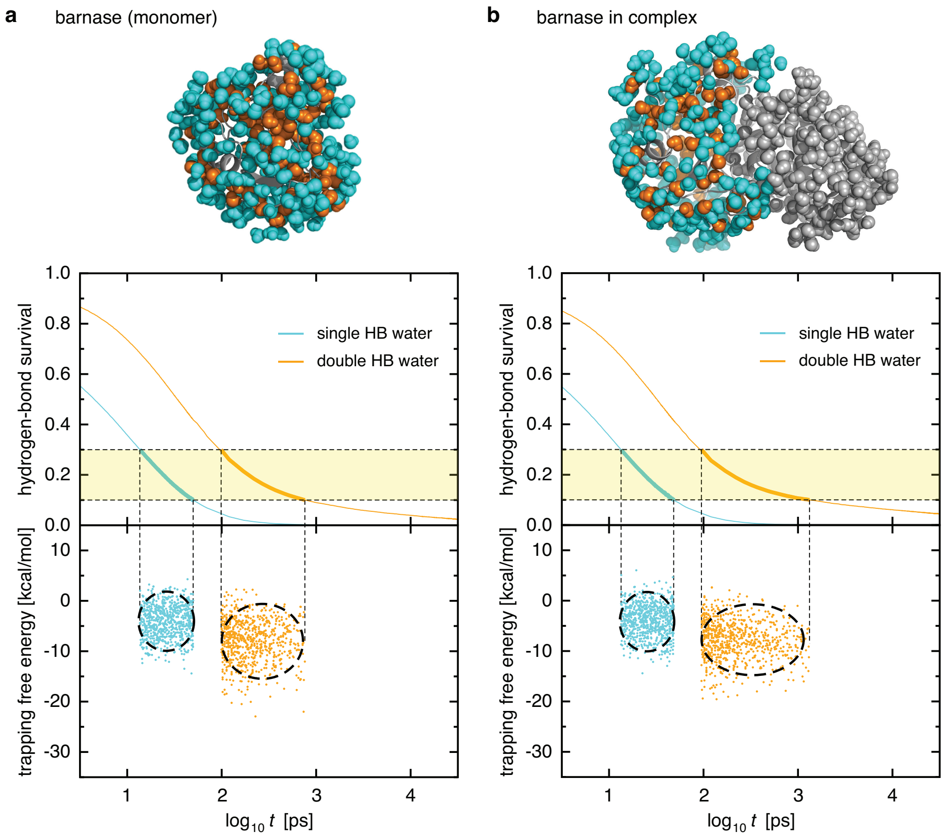

Figure S1: Thermodynamic–dynamic relationship diagram of hydration water for barnase monomer (a) and for barnase in the complex (b). Upper panel: Hydrogen-bond time-correlation functions for single HB water (cyan) and double HB water (orange) versus the logarithmic time axis. Lower panel: Construction of the thermodynamic–dynamic relationship diagram focusing on the time regime (colored by light yellow in the upper panel) where the time-correlation functions decay from 0.3 to 0.1. Scatter plots of the hydrogen-bond survival times and trapping free energies of individual molecules contributing to this relaxation regime are presented. Centers of ellipsoids are determined by the averages, and the width and hight are determined by 3.6 σ (where σ is the standard deviation) along each axis.

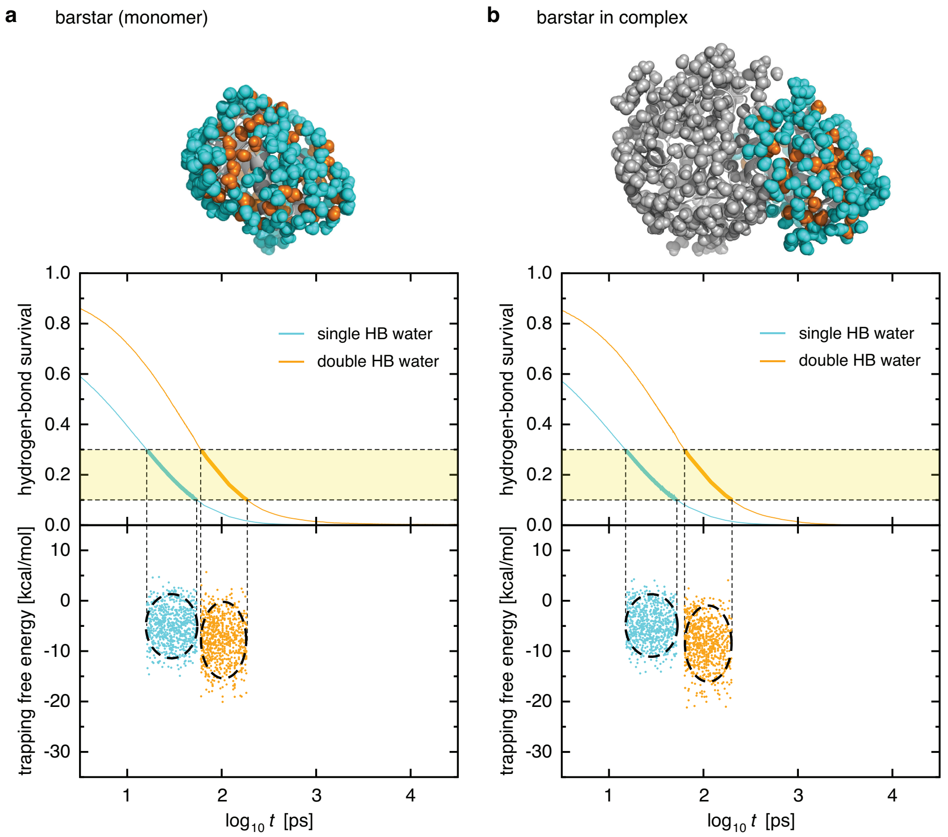

Figure S2: Thermodynamic–dynamic relationship diagram of hydration water for barstar monomer (a) and for barstar in the complex (b). Upper panel: Hydrogen-bond time-correlation functions for single HB water (cyan) and double HB water (orange) versus the logarithmic time axis. Lower panel: Construction of the thermodynamic–dynamic relationship diagram focusing on the time regime (colored by light yellow in the upper panel) where the time-correlation functions decay from 0.3 to 0.1. Scatter plots of the hydrogen-bond survival times and trapping free energies of individual molecules contributing to this relaxation regime are presented. Centers of ellipsoids are determined by the averages, and the width and hight are determined by 3.6 σ (where σ is the standard deviation) along each axis.

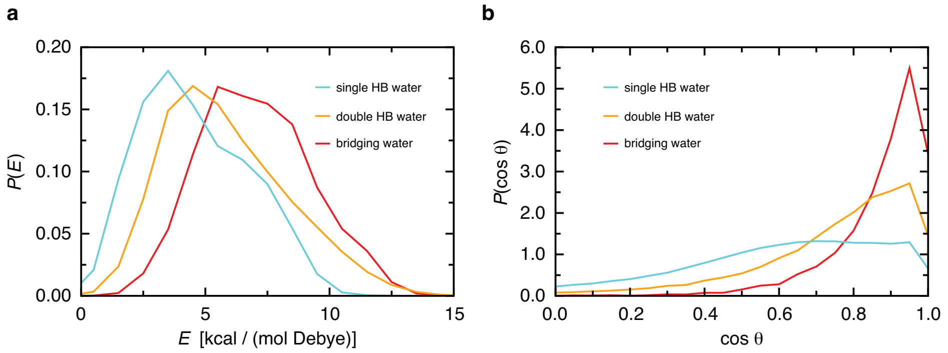

Figure S3: Probability distributions for the magnitude of the electrostatic field E (a) and for $\cos\theta$ with θ denoting the angle between the water's dipole vector and the electrostatic field (b) for single HB water (cyan), double HB water (orange), and bridging water (red).

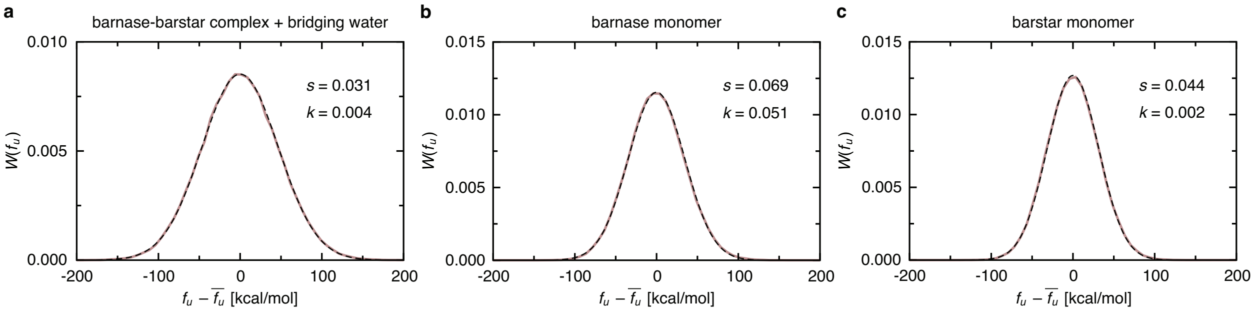

Figure S4: Probability distribution $W(f_u)$ of f_u for the barnase-barstar complex + bridging water (a), barnase monomer (b), and barstar monomer (c) as a function of $f_u - \overline{f_u}$. Dashed curve in each panel denotes the fit by the Gaussian distribution. Closeness to the Gaussian distribution is measured by the skewness (s) and excess kurtosis (k), both of which are zero for the Gaussian distribution.
